# Supplementary material for: CaSPDS, a Spermidine Synthase Gene from Pepper (Capsicum annuum L.), Plays an Important Role in Response to Cold Stress
Source: Int J Mol Sci. 2023 Mar 6;24(5):5013. doi: 10.3390/ijms24055013 (PMC10003509; doi:10.3390/ijms24055013)
Supplement: Supplementary file 1 [file ijms-24-05013-s001.zip › Supplement figures.pdf]

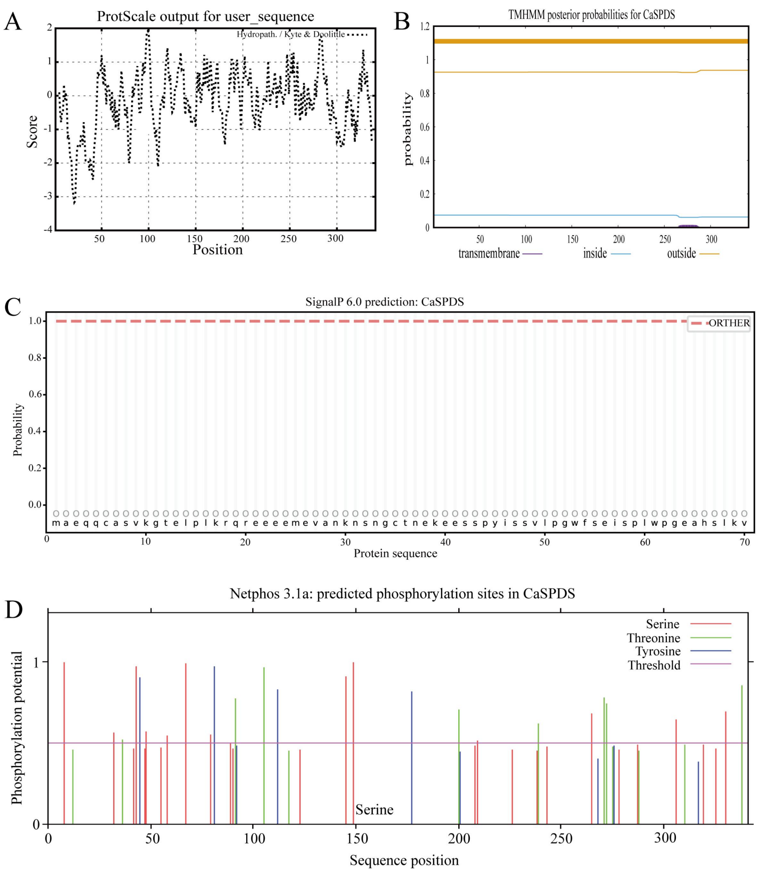

Figure S1. Bioinformatics analysis of the CaSPDS in pepper. (A) Hydrophilicity/hydrophobicity prediction of CaSPDS protein. (B) robability of CaSPDS protein located in/out/across membrane. (C) Signal peptide prediction of CaSPDS protein. (D) Phosphorylation site prediction of CaSPDS protein.

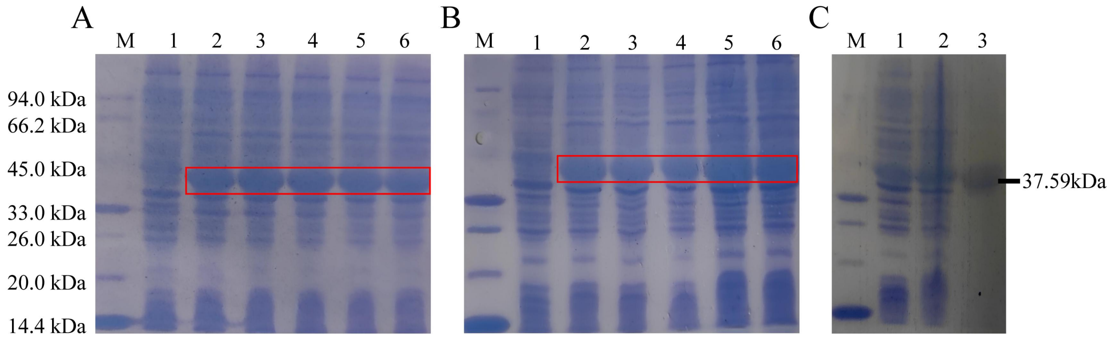

Figure S2. Heterologous expression of CaSPDS protein. (A) Expression of recombinant CaSPDS with different IPTG concentrations. M, protein marker; Lanes 1–6. p-COLD-*CaSPDS* induced by 0, 0.2, 0.4, 0.6, 0.8, and 1 mM IPTG, respectively. (B) Expression of recombinant CaSPDS with different times. M, protein marker; Lanes 1–6. p-COLD-*CaSPDS* induced for 0, 2, 6, 12, 24, 36 h, respectively. (C) Purification of the recombinant protein CaSPDS. M, protein marker; Lane 1: supernatant after pyrolysis; Lane 2: precipitation after pyrolysis; lane 3, purified recombinant protein.

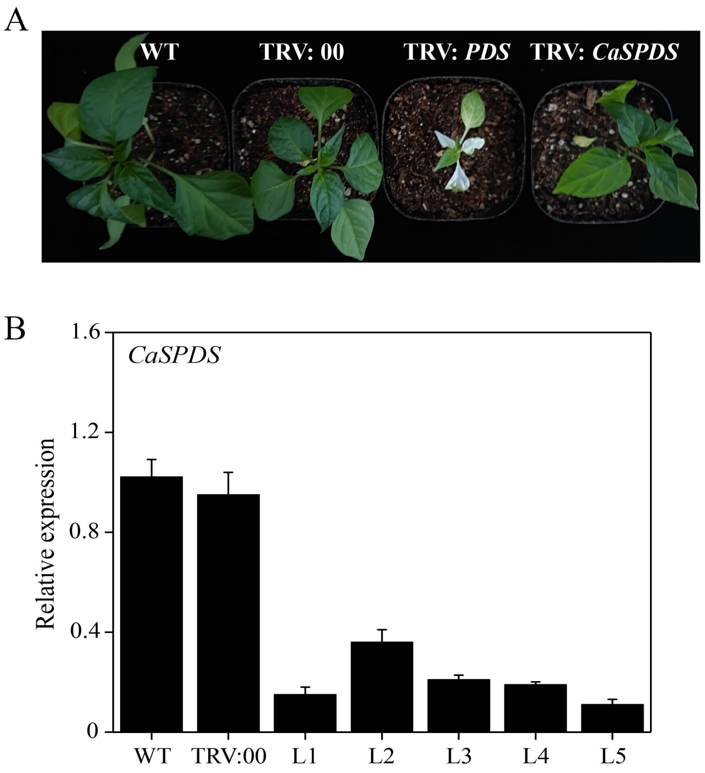

Figure S3. Silencing efficiency of *CaSPDS* expression in pepper seedlings by the VIGS technique. (A) Phenotypes of gene-silenced pepper seedlings 3-4 weeks after inoculation. (B) Analysis of *CaSPDS* expression levels by qRT-CPR after VIGS. *CaUbi3* was used as an international control. WT: Seedlings without bacterial liquid injection; TRV:00, seedlings inoculated with empty vector (negative control); TRV:*PDS*, seedlings of *PDS*-silenced (positive control); TRV2:*CaSPDS*, seedlings of *CaSPDS*-silenced.

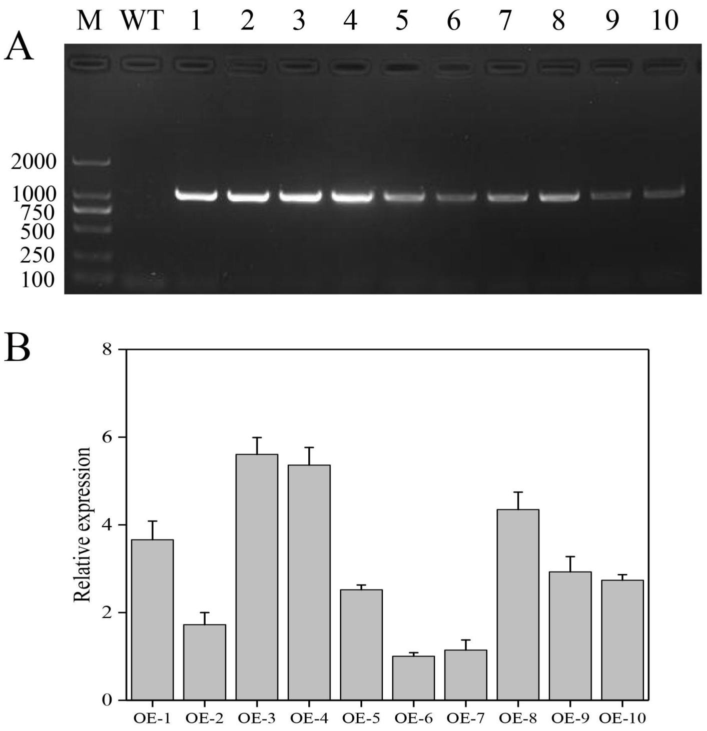

Figure S4. Identification and Expression Level analysis of *CaSPDS* in transgenic *Arabidopsis*. (A) PCR investigation performed to check the 1023 bp coding sequence in the T2 selection stage using the 2,000 bp marker. (B) Relative expression level of *CaSPDS* gene in all transgenic lines. The line with the lowest expression of *CaSPDS* was used as the control, and was set as 1.
