# Supplementary material for: CaSPDS, a Spermidine Synthase Gene from Pepper (Capsicum annuum L.), Plays an Important Role in Response to Cold Stress
Source: Int J Mol Sci. 2023 Mar 6;24(5):5013. doi: 10.3390/ijms24055013 (PMC10003509; doi:10.3390/ijms24055013)
Supplement: Supplementary file 1 [file ijms-24-05013-s001.zip › Supplement tables.pdf]

Table S1. Sequences of the primers used in this study

Primer name	Primer sequences
Q-CaSPDS-F	aggtgcttgttttggatggt
Q-CaSPDS-R	tcctcctccaataaccagca
Q-Actin-F	tgtccatctgctctctgttg
Q-Actin-R	caccccaagcacaataagac
GFP-CaSPDS-F	cagtGGTCTCacaacatggcagagcagcagtgtgcttc
GFP-CaSPDS-R	cagtGGTCTCatacattttcctttggtttcaatcaccctcttggc
CaSPDS-PE-F	cgGAATTCatggcagagcagcagtgt
CaSPDS-PE-R	gacGTCGACtcattttcctttggtttcaaatcaccc
PDS-VI-F	gggGAATTCtgttgtcaaaactccaaggtctgta
PDS-VI-R	gggGGATCCtttctcccacttggttcactcttgt
CaSPDS-VI-F	gggGAATTCcacgttggtgatgatggagttgc
CaSPDS-VI-R	gggGGATCCgtttgttaacagggttcttg
CaSPDS-OE-F	cagtCGTCTCacaacatggcagagcagcagtgtgc
CaSPDS-OE-R	cagtCGTCTCatacatcattttcctttggtttcaa
Q-AtCOR15A-F	gcagatggtgagaaagcgaa
Q-AtCOR15A-R	ggcatccttagcctctcctg
Q-AtRD29-F	gccgagaaacttcagattgg
Q-AtRD29-R	ccattcctcctcctcctttc
Q-AtCOR47-F	cagtgtcggagagtgtggtg
Q-AtCOR47-R	acagctggtgaatcctctgc
Q-AtKIN1-F	tggagctggagcacaaca
Q-AtKIN1-R	gacccgaatcgctacttgttc
AtActin2-F	taacagggagaagatgactcagatca
AtActin2-R	aagatcaagacgaaggatagcatgag

Note: F, forward primer; R, reverse primer; The upper case letters in the primer sequence are restriction endonuclease sites, and the underlined lower case letters are protective bases.
